# Necrolytic Migratory Erythema as the First Manifestation of Functional Conversion in a Glucagonoma: A Case Report

**DOI:** 10.1002/ccr3.71679

**Published:** 2025-12-14

**Authors:** Matheus Felipe Ferreira Aguiar, Jose Jukemura

**Affiliations:** ^1^ Department of Gastroenterology, Hospital das Clínicas, University of São Paulo, School of Medicine, Sao Paulo Brazil

**Keywords:** glucagonoma, necrolytic migratory erythema, neuroendocrine tumor, pancreas

## Abstract

Necrolytic migratory erythema is a characteristic but often underrecognized manifestation of glucagonoma. Its timely identification can lead to early diagnosis and curative surgery of pancreatic neuroendocrine tumors that initially present as clinically silent lesions.

## 1 Introduction

Pancreatic neuroendocrine tumors (PNETs) are a rare and heterogeneous group of tumors, comprising less than 5% of all pancreatic malignancies. These tumors are subclassified according to the specific hormones they secrete. Among them, glucagonoma is an exceptionally rare variant, with an estimated annual incidence of approximately 1 case per 20 million individuals [1]. Glucagonomas arise from islet alpha cells of the pancreas, with a predilection for the tail of the pancreas, likely due to the increased concentration of islet cells in this region, and more than 50% of the patients present metastasis at the diagnosis [2].

The diagnosis of glucagonoma is often made in the context of glucagonoma syndrome, which is characterized by the autonomous secretion of glucagon by the tumor. The clinical manifestations of this syndrome are diverse, including necrolytic migratory erythema (NME), diabetes mellitus, unintentional weight loss, deep vein thrombosis, neuropsychiatric abnormalities, and gastrointestinal symptoms such as diarrhea [3].

We report a case of a 74‐year‐old male with a neuroendocrine tumor located in the tail of the pancreas, asymptomatic at the time of diagnosis. During clinical follow‐up, the patient developed skin lesions consistent with necrolytic migratory erythema. He underwent surgical intervention, with immunohistochemical findings consistent with glucagonoma, resulting in the remission of the lesions and a subsequent prolonged disease‐free period.

## 2 Case History/Examination

A 74‐year‐old male was referred for evaluation following the incidental identification of a small hypervascular pancreatic nodule, measuring 8 mm, located at the body‐tail junction of the pancreas, on abdominal magnetic resonance imaging (MRI). Initially, the patient was asymptomatic, with no clinical signs suggestive of pancreatic involvement. His medical history included well‐controlled hypertension treated with indapamide, hypothyroidism managed with levothyroxine, type 2 diabetes mellitus controlled with metformin, and glaucoma under treatment with dorzolamide. Family history was notable for gastric cancer in both his father and paternal grandfather. Physical examination revealed no significant abnormalities.

Laboratory investigations were within normal limits, and both upper gastrointestinal endoscopy and colonoscopy showed no significant abnormalities. Given the imaging characteristics and absence of clinical symptoms, the lesion was presumed to be a non‐functioning pancreatic neuroendocrine tumor (PNET) measuring less than 1 cm. A conservative management approach with clinical and radiological surveillance was initiated.

**FIGURE 1:**
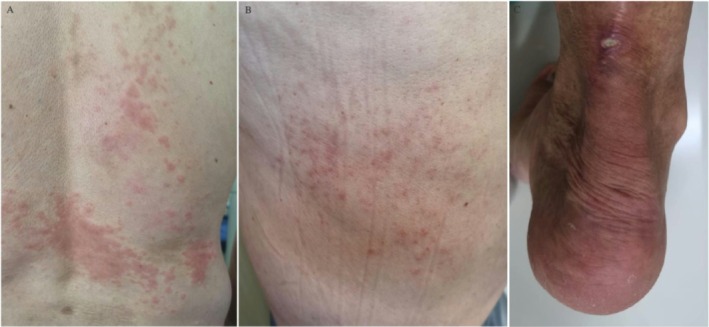
Initial presentation of skin lesions, characterized by erythematous plaques (A) and papules (B), predominantly localized to the dorsal region, along with an ulcerated lesion around the ankle (C).

Over a 30‐month follow‐up period, the patient remained asymptomatic, and the lesion demonstrated radiological stability. However, at 35 months, he developed erythematous plaques and confluent papules, predominantly on the back and lower limbs (Figure 1A,B), some of which gradually progressed to ulcerated lesions (Figure 1C). At that time, plasma glucagon levels were elevated at 222 μg/dL (reference range: < 150 μg/dL). A PET‐CT scan revealed no evidence of metastatic disease.

## 3 Differential Diagnosis

At this time, given the lesions observed, multiple diagnostic hypotheses were formulated, including a range of dermatologic and systemic conditions that may present with erythematous, erosive, or ulcerated cutaneous lesions. Among the primary mimickers are psoriasiform dermatoses, eczema, pemphigus foliaceus, pellagra, zinc deficiency, and necrolytic acral erythema. Additionally, systemic diseases such as celiac disease, Crohn's disease, hepatic cirrhosis, pancreatitis, and bronchogenic carcinoma were also considered. However, the presence of hyperglucagonemia in conjunction with imaging findings of a pancreatic mass supports a definitive diagnosis.

**FIGURE 2:**
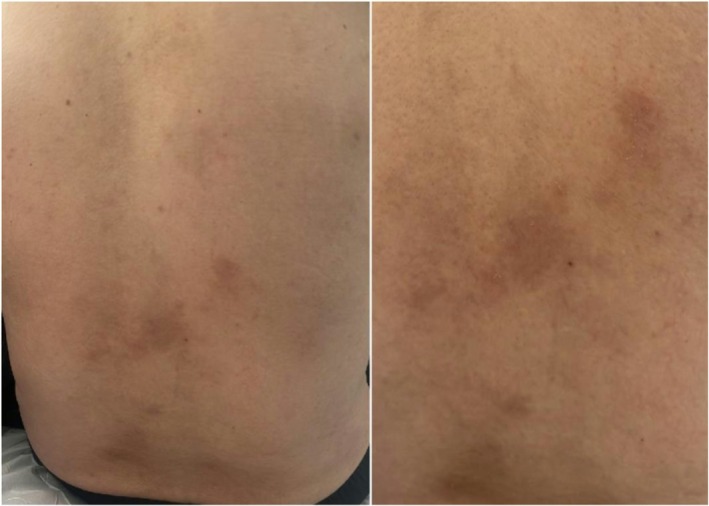
Appearance of the lesions following resection of the glucagonoma, showing a pattern of hyperpigmentation, 3 months post‐surgery.

In light of the clinical, laboratory, and imaging findings, the leading diagnosis was a pancreatic neuroendocrine tumor that had initially appeared non‐functional but subsequently demonstrated glucagon hypersecretion, clinically manifesting as necrolytic migratory erythema. Surgical intervention was indicated. The patient underwent distal pancreatectomy with splenectomy, with no intraoperative complications and a favorable postoperative course. The cutaneous lesions regressed within weeks following tumor resection, evolving into areas of post‐inflammatory hyperpigmentation (Figure 2). Serum glucagon levels decreased to within the normal range, reaching 169 μg/dL postoperatively.

## 4 Conclusion and Results (Outcome and Follow‐Up)

**FIGURE 3:**
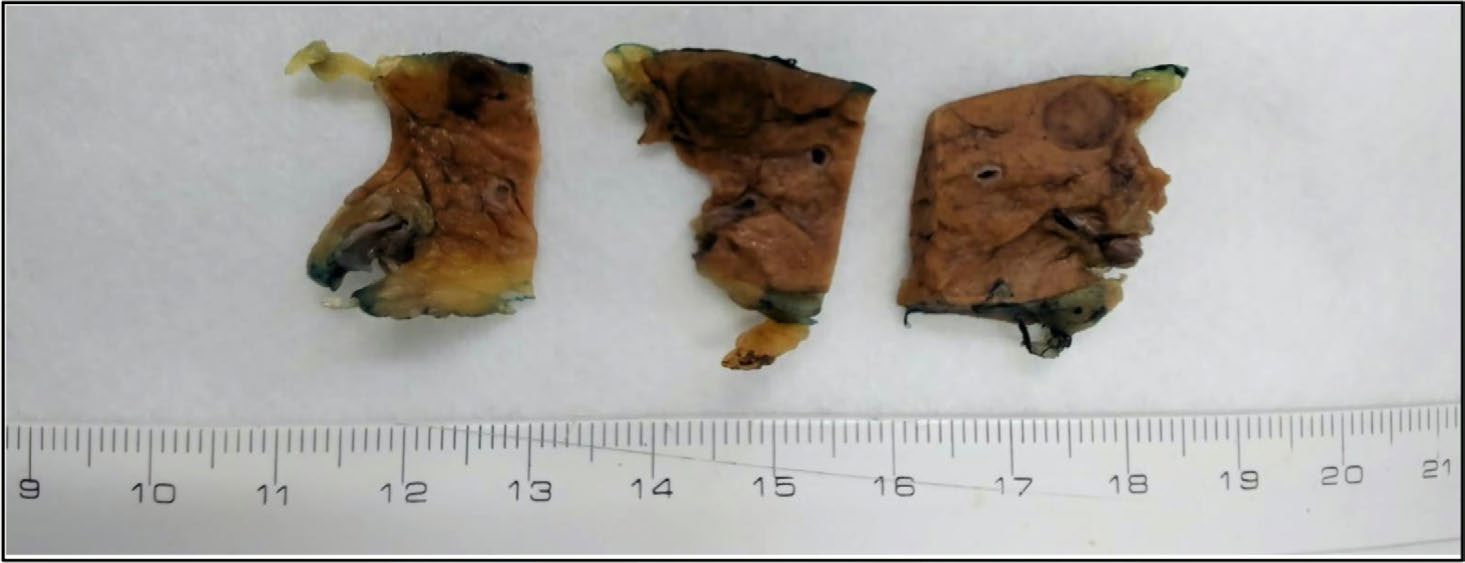
Postoperative specimen.

**FIGURE 4:**
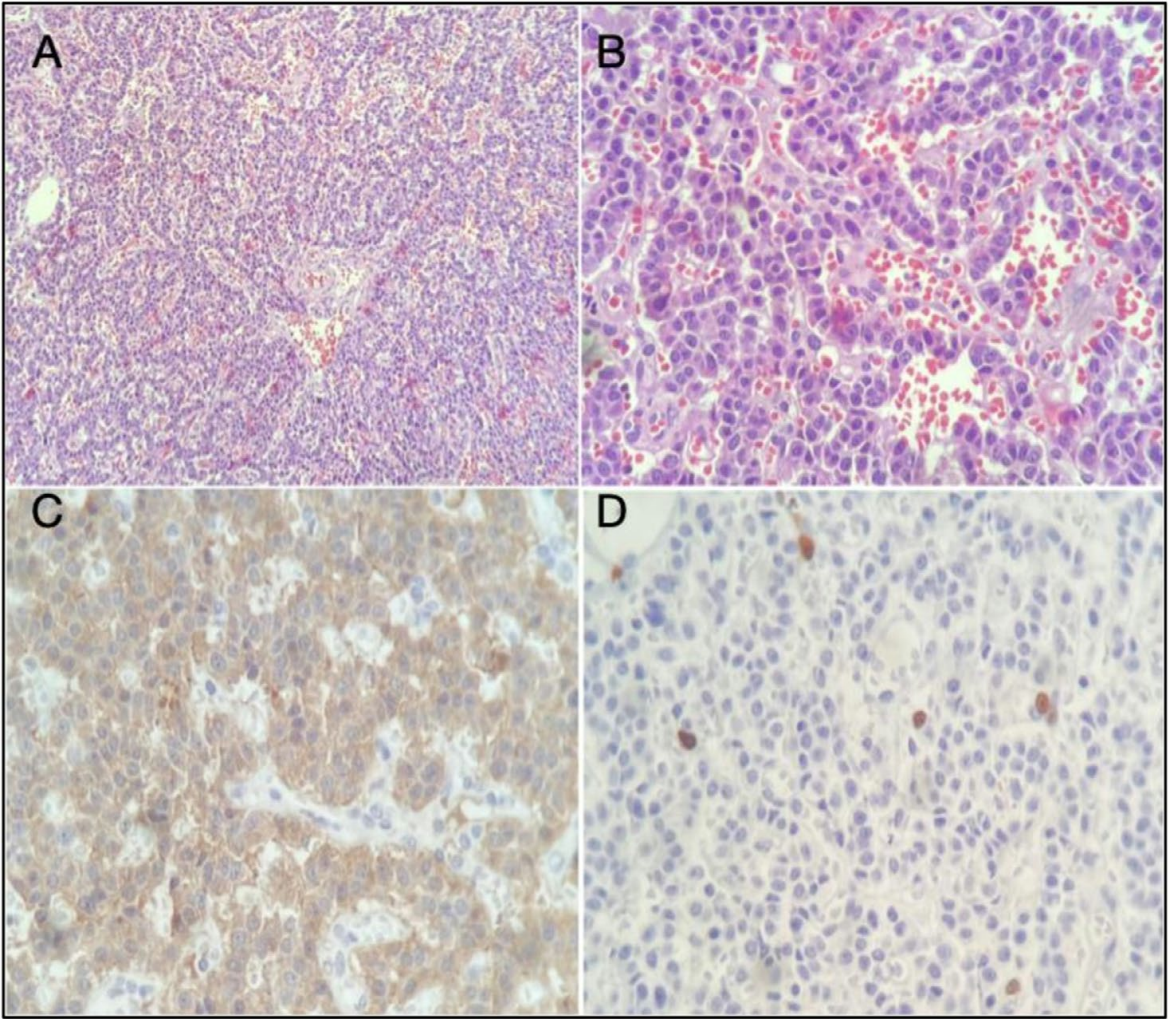
(A and B) Hematoxylin and eosin staining at low and high magnification showing a well‐differentiated neuroendocrine tumor arranged in solid nests. The tumor cells are monotonous, with moderate cytoplasm and characteristic stippled “salt‐and‐pepper” chromatin. (C) Immunohistochemistry demonstrates diffuse cytoplasmic positivity for synaptophysin. (D) Ki‐67 immunostaining reveals a low proliferative index of 1%, supporting the diagnosis of a well‐differentiated grade 1 glucagonoma.

Histopathological analysis confirmed a well‐differentiated (G1) pancreatic neuroendocrine tumor measuring 0.8 × 0.7 × 0.5 cm (Figure 3), restricted to the pancreatic parenchyma, with a mitotic index of < 1 mitosis/mm^2^, no necrosis, no vascular or perineural invasion, and negative surgical margins. None of the twelve examined peripancreatic and perisplenic lymph nodes showed tumor involvement, corresponding to a pathological stage of pT1. Additionally, a lesion compatible with pancreatic intraepithelial neoplasia with low‐grade dysplasia was identified within the specimen, with preserved resection margins. Immunohistochemistry revealed strong positivity for chromogranin A, synaptophysin, cytokeratins 8 and 18, a Ki‐67 proliferation index of 1%, and positive glucagon expression in the distal segment of the pancreas (Figure 4).

**FIGURE 5:**
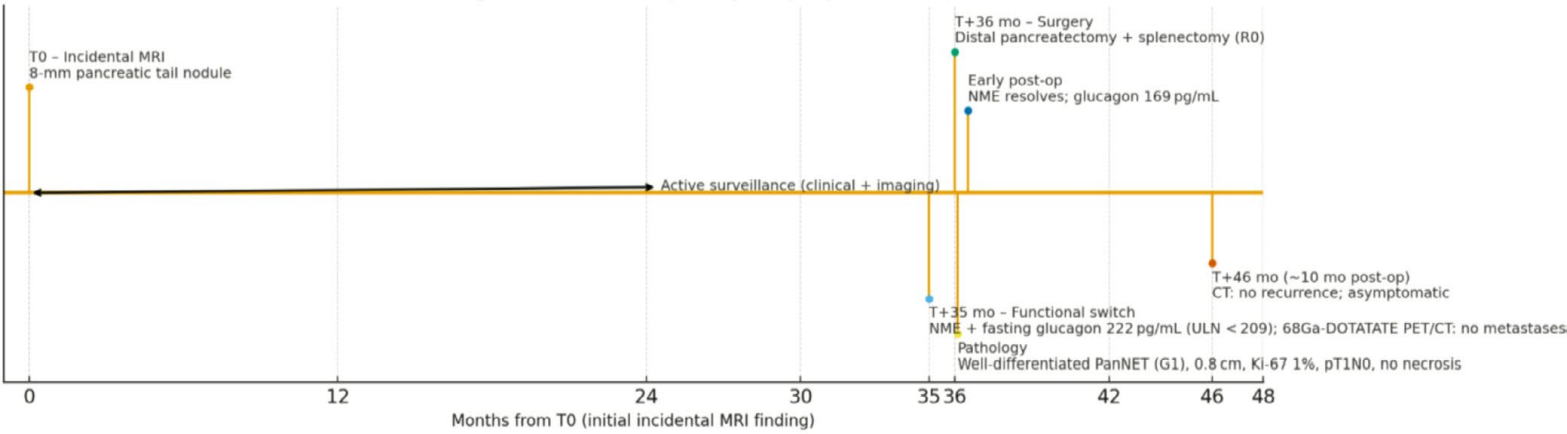
Case timeline. Months from T0 (initial incidental MRI finding). The patient had an 8‐mm pancreatic tail nodule under active surveillance (T0–T + 30 months). At T + 35 months, necrolytic migratory erythema (NME) appeared with elevated fasting plasma glucagon (222 pg/mL; ULN < 209 pg/mL) and 68Ga‐DOTATATE PET/CT showed no metastases. Distal pancreatectomy with splenectomy at T + 36 months achieved R0 resection. Pathology: Well‐differentiated PanNET (G1), 0.8 × 0.7 × 0.5 cm, Ki‐67 1%, pT1N0, no necrosis, negative margins. NME resolved within weeks; postoperative glucagon was 169 pg/mL. At ~10 months post‐op (T + 46 months), CT showed no recurrence.

Ten months after tumor resection, the patient is clinically stable, with no new dermatological lesions. A follow‐up abdominal CT scan showed no abnormalities. The chronology and evolution of the presented case are shown in Figure 5 (timeline).

## 5 Discussion

Pancreatic neuroendocrine tumors (PNETs) represent a diverse group of tumors originating from the islet cells of the pancreas. While the annual incidence of PNETs is typically less than one case per 100,000 individuals, the overall incidence has been increasing [4, 5]. This rise is likely attributed to advances in imaging technologies, such as cross‐sectional imaging and endoscopic ultrasonography, along with greater recognition by healthcare providers. Although PNETs are often considered less aggressive than pancreatic ductal adenocarcinoma (PDAC), their potential to metastasize to regional lymph nodes and distant sites remains a concern. Additionally, due to their anatomical location and size, PNETs can cause local effects [6].

These tumors are usually classified based on their functional activity and grade. Functional pancreatic neuroendocrine tumors (F‐PNETs) are characterized by hormone secretion, which results in specific clinical syndromes. In contrast, nonfunctional pancreatic neuroendocrine tumors (NF‐PNETs) do not secrete hormones and are often asymptomatic. Nonfunctional tumors represent the majority of PNET cases, comprising 60%–90%. Functional tumors tend to have a more favorable prognosis compared to nonfunctional ones, possibly because the hormone production in functional tumors leads to paraneoplastic syndromes, which can aid in earlier detection and provide clearer indicators of tumor progression [5, 7].

In 1942, Becker et al. [8] were the first to report the association between a cutaneous eruption and pancreatic cancer, observed in a patient with an alpha‐cell pancreatic tumor. Subsequently, in 1966, McGavran et al. [9] described the clinical presentation of a glucagon‐secreting pancreatic tumor, which was also associated with diabetes mellitus and bullous dermatitis. The term necrolytic migratory erythema (NME) was first introduced in 1973 by Wilkinson [10] to refer to the characteristic dermatologic manifestation. In 1974, Mallinson et al. [11] delineated the glucagonoma syndrome, which is characterized by the triad of NME, weight loss, and hyperglucagonemia, along with other associated systemic symptoms.

Glucagonomas are extremely rare pancreatic neuroendocrine tumors (pNETs), with an incidence of about one in 20 million people [12]. They are typically diagnosed between the fifth and sixth decades of life, affecting both men and women equally. Most cases are sporadic, with less than 3% associated with multiple endocrine neoplasia type 1 (MEN1). These tumors are often located in the pancreatic tail in 87% of cases, and over 50% of patients present with metastatic disease at diagnosis, underlining the need for early detection [6, 13]. A study conducted in the United States over 28 years identified 2705 cases of pancreatic neuroendocrine tumors, with glucagonomas accounting for only 1.3% of these neoplasms [14]. Another report found a glucagonoma incidence of 2% among 1310 pancreatic neuroendocrine tumors during the same period. Additionally, a separate study observed that approximately 7% of 340 pancreatic neuroendocrine tumors were glucagonomas [15]. These findings underscore the rarity of glucagonomas among pancreatic neuroendocrine tumors.

Glucagonoma can manifest clinically as a paraneoplastic phenomenon termed “glucagonoma syndrome” and is characterized by a spectrum of symptoms **such** as necrolytic migratory erythema (NME), hyperglucagonemia, diabetes mellitus, anemia, weight loss, glossitis, steatorrhea, diarrhea, venous thrombosis, and neuropsychiatric disturbances. However, a significant number of patients with glucagonoma have no clinical symptoms, and the clinical presentation is not uniform [8, 9, 10, 11, 12].

Necrolytic migratory erythema (NME) is a defining feature of glucagonoma, present in nearly 70% of patients with glucagonoma syndrome, and often serves as the first clinical sign of the disease [16]. This rare skin condition is commonly associated with pancreatic islet cell tumors, and its early recognition can lead to a timely diagnosis, as highlighted in various case reports. In fact, NME can sometimes be the only presenting symptom of glucagonoma syndrome, underscoring the importance of early detection, which facilitates appropriate intervention and management [17]. In our case report, NME was the sole clinical manifestation of glucagonoma.

The typical skin lesions of necrolytic migratory erythema (NME) start as well‐defined red papules or plaques, which then progress to vesicles and soft blisters. Over time, these lesions undergo central necrosis, leading to crusting, ulceration, and eventual post‐inflammatory hyperpigmentation. The lesions mainly affect areas prone to friction, such as the groin, buttocks, genital region, and lower legs. They can also appear around the mouth, on the trunk, and on the face [16, 17]. NME lesions follow a cyclical pattern, alternating between flare‐ups and periods of remission, often with no clear trigger. The Koebner phenomenon, where lesions develop at sites of trauma, is commonly seen [18]. As shown in the figures, different stages of lesion development were observed in our report, including erythematous plaques, ulcerated lesions, and hyperpigmentation in the later stage.

Diagnosing glucagonoma based solely on the presence of erythema can be difficult, as necrolytic migratory erythema (NME) is frequently mistaken for various other dermatological conditions. These include measles vasculitis, eosinophilic cellulitis characterized by edematous erythema, and eczema with erosive features, such as seen in pemphigus. Additionally, several other medical conditions, including liver disease, inflammatory bowel disease, pancreatitis, celiac disease, Crohn's disease, hepatic cirrhosis, and other cancers like bronchial carcinoma, can also exhibit NME. Collectively, these conditions are often referred to as pseudoglucagonoma syndrome [16].

Several theories have been proposed regarding the pathogenesis of necrolytic migratory erythema (NME). Hyperglucagonemia appears to play a central role, as normalizing glucagon concentrations through tumor resection typically leads to rapid improvement in the skin lesions. Elevated glucagon levels induce a catabolic state, resulting in deficiencies of amino acids, essential fatty acids, and minerals that are critical for skin integrity. These deficiencies disrupt epidermal regeneration, contributing to necrosis and epidermal breakdown. However, abnormal glucagon levels alone may not fully explain the skin findings, as elevated glucagon is also observed in conditions such as trauma, burns, diabetic ketoacidosis, starvation, and cirrhosis, none of which are typically associated with the characteristic rash [19].

Necrolytic migratory erythema (NME) demonstrates distinct histological characteristics, including paleness and spongiosis of the upper layer of the epidermis. A perivascular infiltrate of lymphocytes and histiocytes is commonly observed, along with necrotic keratinocytes, which can lead to erosions, crusting, and scaling. The most specific feature on skin histological examination is superficial epithelial necrosis. In some cases, apoptotic keratinocytes may be highlighted by staining with immunoglobulins, fibrinogen, and C3 on direct immunofluorescence. This histological picture is similar to other deficiency states, such as pellagra, zinc deficiency, and necrolytic acral erythema. Skin biopsy in cases of NME demonstrates superficial necrolysis with separation of the outer layers of the epidermis and perivascular infiltration by lymphocytes and histiocytes. These findings are crucial for the diagnosis and understanding of the pathology associated with NME [20].

The diagnosis of glucagonoma (GCGN) involves a combination of specific clinical manifestations and essential imaging tools. Computed tomography (CT) and magnetic resonance imaging (MRI) are crucial for studying the pancreas, allowing precise tumor localization, assessment of its relationship with adjacent organs, and identification of metastases. Measurement of plasma glucagon levels is decisive for diagnosis, with levels above 500 ng/L considered indicative of GCGN. Selective visceral angiography is considered the gold standard for locating and diagnosing these tumors due to their characteristic hypervascularization, although some cases may present mild contrast enhancement [18]. Additionally, positron emission tomography (PET) and octreotide scintigraphy are of great importance, as most glucagonomas have receptors for somatostatin [16, 21].

The treatment of glucagonoma primarily involves surgical intervention, which can be performed through conventional open surgery or minimally invasive laparoscopic surgery. In cases of early diagnosis, surgical resection may be curative. For patients with metastatic liver disease, several therapeutic options are available, including hepatic artery embolization, which may involve chemoembolization or microsphere radioembolization, both aimed at reducing the blood supply to liver metastases. Liver transplantation has also been explored as a treatment for patients with unresectable liver metastases, with studies suggesting favorable outcomes in some cases. In addition, therapies such as cryoablation and metastasectomy may be considered for patients with disseminated liver disease. For those with contraindications to surgery, chemotherapy using agents like doxorubicin and streptozotocin may be utilized, as they selectively damage pancreatic islet cells. Moreover, somatostatin analogs, peptide receptor radionuclide therapy (PRRT), and molecular targeted drugs have shown effectiveness in controlling tumor growth and alleviating clinical symptoms. Given the weak response of glucagonoma to chemotherapy, surgical removal remains the most effective approach to treatment, especially when aimed at lowering serum glucagon levels and preventing further disease progression [12, 13].

## 6 Conclusion

The present case illustrates how necrolytic migratory erythema, despite its rarity, can serve as a key clinical marker for the diagnosis of glucagonoma. Given the nonspecific and often indolent nature of pancreatic neuroendocrine tumors, heightened clinical awareness of paraneoplastic dermatologic syndromes is essential for early recognition. Surgical resection remains the cornerstone of treatment and can be curative when performed before metastatic progression. Ultimately, this case underscores the diagnostic value of cutaneous findings in systemic diseases and reinforces the need for multidisciplinary vigilance in the management of rare functional neuroendocrine tumors.

## Author Contributions

**Matheus Felipe Ferreira Aguiar:** data curation, methodology, writing – original draft, writing – review and editing. **Jose Jukemura:** formal analysis, funding acquisition, methodology, project administration, supervision, validation, visualization.

## Funding

The authors have nothing to report.

## Consent

A written informed consent was obtained from the patient to publish this case report in accordance with the journal's patient consent policy.

## Conflicts of Interest

The authors declare no conflicts of interest.

## Data Availability

The data that support the findings of this study are available from the corresponding author upon reasonable request.
